# Impact of the severity of obesity on microalbuminuria in obese normotensive nondiabetic individuals

**DOI:** 10.12861/jrip.2015.08

**Published:** 2015-06-01

**Authors:** Farzanehsadat Minoo, Mitra Mahdavi-Mazdeh, Mohamad-Reza Abbasi, Shahram Sohrabi

**Affiliations:** ^1^Nephrology Research Center, Tehran University of Medical Sciences, Tehran, Iran; ^2^Iranian Tissue Bank and Research Center, Tehran University of Medical Sciences, Tehran, Iran; ^3^Nephrology Research Center, Tehran Municipally, Tehran, Iran

**Keywords:** Microalbuminuria, Obesity, Body mass index, Diabetes Hypertension

## Abstract

**Introduction:** Microalbuminuria has been now recognized as the most important risk factor for the increased morbidity and mortality in the obese population.

**Objectives:** We aimed to know whether severity of obesity is associated with the presence of renal injury while microalbuminuria acts, independent of other risk factors as hypertension and diabetes mellitus.

**Patients and Methods:** The current cross-sectional study was conducted on consecutive obese normotensive nondiabetic individuals. Two groups of adult individuals were selected as controls comprised of 161 obese adults with body mass index (BMI) 30-35 kg/m^2^ and 25 very obese adults as cases with BMI more than 35 kg/m^2^. Microalbuminuria was defined as abnormal urinary albumin to creatinine ratio (UACR) more than 30 mg/g of creatinine.

**Results:** No significant differences in serum creatinine level, urinary albumin concentration, as well as UACR between obese and very obese individuals was seen. Using Pearson correlation coefficient analysis, no significant correlation was observed between BMI and parameters of renal function. Microalbuminuria was more prevalent in very obese individuals compared with obese group (24.0% versus 9.9%, *P* = 0.043) in univariate analysis.

**Conclusion:** Severe obesity compared with milder obesity status cannot predict the occurrence of increased urinary albumin excretion and microalbuminuria.

**Implication for health policy/practice/research/medical education:**

Severe obesity compared with milder obesity status cannot predict the occurrence of increased urinary albumin excretion and microalbuminuria.

## Introduction

With the rapidly growth of the obesity epidemic whole of the world, a better understanding of the risk factors for the complications of obesity is critical. Nowadays, microalbuminuria has been now recognized as the most important risk factor for the increased morbidity and mortality in the obese population (1-4). In this parallel, central obesity has received more attention as a potential risk factor for renal insufficiency in nondiabetic normotensive subjects (5,6). The mechanisms of this association are now unclear and might be mediated by adipogenic inflammation as well as endothelial dysfunction giving microalbuminuria. Besides, obesity is associated with subtle effects in the decline of kidney function and low-grade albuminuria (7-10), and this event potentially results in appearance and progression of cardiovascular diseases in obese patients (11). Several key early studies established a potential important relationship between obesity and microalbuminuria. However, association between the severity of obesity and appearance of microalbuminuria is unknown.

## Objectives

We aimed to know whether severity of central obesity is associated with the presence of renal injury while microalbuminuria acts, independent of other risk factors like blood pressure and fasting blood glucose.

## Patients and Methods

The current cross-sectional study was conducted on consecutive obese individuals (body mass index [BMI] ≥ 30 kg/m^2^) and older than 18 years attending the outpatient health clinic at the Tehran municipality in 2011. Exclusion criteria were: history of diabetes mellitus, hypertension, hyperlipidemia, cigarette smoking, family history of cardiovascular disease, any renal or liver conditions, urinary tract infections, or menstruation phase of the menstrual cycle. The study was conducted after taking the required ethical clearance from the ethics committee of Tehran University of Medical Sciences and all patients who signed the informed consent participated in the study.

Anthropometric index of BMI (body weight in kg/height in m^2^) was recorded and two groups of adult individuals were selected. Group I as controls comprised of 161 obese adults with BMI 30-35 kg/m^2^ (obese group) and group II had 25 very obese adults as cases with BMI > 35 kg/m^2^. Baseline data were collected by face to face interviewing including demographics and educational level. Weight was measured using a balance standard scale and standing height was measured using a single stedeometer. Blood pressure was measured using a standard mercury sphygmomanometer. Around 5 ml of spot urine sample in a sterile container was collected for determination of spot urinary albumin excretion and urinary creatinine. Estimation of urinary albumin was carried out by immunonephelometric assay, and urinary creatinine was estimated by automated enzymatic assay. Urinary albumin excretion was calculated as urinary albumin creatinine ratio (UACR). UACR was calculated as milligram of albumin/g of creatinine. Microalbuminuria was defined as abnormal UACR (more than 30 mg/g). Blood samples after 10-12 hours of overnight fasting was collected for estimation of fasting blood glucose and serum creatinine. Blood glucose was assayed by glucose oxidase assay kit (Pars Azmoon Ltd, Iran) and serum creatinine was estimated by automated enzymatic assay.

### Ethical issues

The research followed the tenets of the Declaration of Helsinki; informed consent was obtained, and the research was approved by ethics committee of Tehran University of Medical Sciences.

### Statistical analysis

Results were reported as mean ± standard deviation (SD) for the quantitative variables and percentages for the categorical variables. The groups were compared using the *t* test for the continuous variables and the chi-square test (or Fisher exact test if required) for the categorical variables. Correlations between the quantitative variables were examined by the Pearson correlation coefficient test. Predictors exhibiting a statistically significant relation with microalbuminuria were taken for multivariate logistic regression analysis to investigate their independence as predictors. Odds ratio (OR) and 95% CI were calculated. This study was done with the power of 80%. *P* values of 0.05 or less were considered statistically significant. All the statistical analyses were performed using SPSS version 13.0 (SPSS Inc., Chicago, IL, USA) and SAS version 9.1 for Windows (SAS Institute Inc., Cary, NC, USA).

## Results

Totally, 186 obese individuals were studied with a mean age of 30.4 ± 10.7 years that among them 15.5% suffered from severe obesity (BMI > 35 kg/m^2^). As shown in  Table 1,  2 groups with obesity or severe obesity were similar in terms of gender, mean age, educational level, and diastolic blood pressure, while very obese group had significantly higher fasting blood sugar and systolic blood pressure. Regarding criteria of renal function, there were no significant differences in serum creatinine level, urinary creatinine level, urinary albumin concentration, as well as urinary albumin to creatinine ratio (Table 2) (*P *> 0.05). Also, using Pearson correlation coefficient analysis ( Table 3), no significant correlation was observed between BMI and parameters of renal function (*P *> 0.05). As we defined microalbuminuria as urinary albumin to creatinine ratio ≥ 30 mg/g, the overall prevalence of microalbuminuria was 11.8%. Microalbuminuria was more prevalent in very obese compared with obese group (24.0% versus 9.9%, *P* = 0.043). However with the presence of cofounders in a multivariable regression model, severe obesity could not predict appearance of microalbuminuria (OR = 1.124, *P *= 0.141) ( Table 4).

**Table 1:** Baseline characteristics of the study groups

**Characteristics**	**Total (n = 186)**	**Obese group (n = 161)**	**Very obese group (n = 25)**	***P*** **-value**
Male gender	11 (5.9)	11 (6.8)	0 (0.0)	0.365
Age (year)	30.4/10.7	39.0/10.5	41.8/12.0	0.223
Weight (kg)	86.5/8.2	84.9/7.1	96.6/7.6	<0.001
BMI (kg/m^2^)	32.8/2.6	32.0/1.3	38.1/2.9	<0.001
Education level				0.469
Primary	83 (44.6)	69 (42.9)	14 (56.0)	
Secondary	75 (40.3)	67 (41.6)	8 (32.0)	
College degree	28 (15.1)	25 (15.5)	3 (12.0)	
Fasting blood sugar (mg/dl)	96.0/23.9	94.3/21.4	106.8/34.7	0.015
Systolic blood pressure (mm Hg)	111.6/7.9	111.1/8.0	114.4/7.1	0.042
Diastolic blood pressure (mm Hg)	76.4/4.8	76.4/4.8	76.4/4.9	0.998

Abbreviation: BMI, body mass index.

**Table 2:** Parameters of renal function in the study groups

**Characteristics**	**Total (n = 186)**	**Obese group (n = 161)**	**Very obese group (n = 25)**	*** P *** **-value**
Serum creatinine (mg/dl)	0.83/0.11	0.83/0.10	0.83/0.12	0.941
Urinary creatinine (mg/dl)	0.11/0.05	0.12/0.05	0.10/0.04	0.062
Urinary albumin (mg/dl)	2.4/4.0	2.4/4.3	2.1/0.50	0.286
UACR (mg/g)	23.4/31.8	23.1/33.4	25.5/18.9	0.613

Abbreviation: UACR, urinary albumin creatinine ratio.

**Table 3:** Correlation between BMI and renal function indices

**Index**	**Correlation index (r)**	*** P *** **-value**
BMI and serum creatinine	0.074	0.315
BMI and urinary creatinine	-0.100	0.176
BMI and urinary albumin	-0.001	0.992
BMI and UACR	0.065	0.376

Abbreviations: UACR, urinary albumin creatinine ratio; BMI, body mass index.

**Table 4:** Main determinants of microalbuminuria in the study population

**Variable**	**Multivariable,** *** P *** **-value**	**OR**	**95% CI**
Age	0.736	0.993	0.950–1.037
BMI	0.141	1.124	0.962–1.315
Systolic blood pressure	0.907	1.004	0.943–1.068
Diastolic blood pressure	0.625	0.976	0.884–1.077
Serum creatinine	0.448	0.176	0.002–15.750
Fasting blood sugar	0.630	1.004	0.987–1.022

Abbreviations: OR; odds ratio; BMI, body mass index.

## Discussion

Studies focused on microalbuminuria in nondiabetic non-hypertensive subjects especially in obese ones are limited. Some available studies suggested the role of microalbuminuria for predicting poor outcome of patients with cardiovascular diseases even after adjusting cardiovascular disease risk factors (12). To the best of our knowledge, the current study was the first that evaluate the impact of obesity severity on the presence of microalbuminuria. We showed that severe obesity defined as BMI > 35 kg/m^2^ is not a risk factor for increased urinary albumin excretion in nondiabetic normotensive obese individuals. On the other hand, although obesity might be a risk factor for appearance of microalbuminuria, the rate of this phenomenon is similar in obese and severe obese ones. In fact, severe obesity compared with milder obesity status could not predict the occurrence of increased urinary albumin excretion. Some studies showed that microalbuminuria was associated with obesity in the presence of endothelial dysfunction (13). Valensi et al (14) reported that daily albumin excretion in urine was significantly higher in obese people than in lean people. In their study, the prevalence of microalbuminuria was found to be increased in nondiabetic obese people. Besides, some others obtained contrary findings. In Cubeddu et al (15) study, no relationship between abdominal obesity and microalbuminuria was detected. Also, Hoffmann et al (16) found no different levels of albuminuria in lean, overweight and obese glucose tolerant subjects. Moreover, in Yesim et al (17) study, microalbuminuria was not detected in obese women without diabetes and/or hypertension and urinary albumin excretion was similar in obese and lean women. Recent clinical studies suggest that the adipocyte hormone adiponectin may play a key role in the development of obesity-related albuminuria. Additionally, investigations with the adiponectin knockout mouse indicate that adiponectin can regulate podocyte function and thus contribute to the initial development of albuminuria (18). Future studies to examine these biological processes are needed to confirm these mechanisms involved in the relation between obesity and microalbuminuria especially in nondiabetic normotensive patients.

Although we included nondiabetic normotensive individuals, it is interesting that the elevation of systolic blood pressure (in normal range) associated with microalbuminuria in severe obesity (*P* = 0.042). When intra-abdominal fat mass and total body fat mass were assessed by whole-body dual energy x-ray absorptiometry scanning and abdominal computed tomography scanning, no correlation was observed between visceral fat accumulation and urinary albumin excretion in young, normal glucose tolerant, female and male Caucasians (19). These observations, together with our findings indicate that abdominal obesity or its severity is not a determinant per se of increased urinary albumin excretion (18-20).

## Conclusion

Severe obesity compared with milder obesity status cannot predict the occurrence of increased urinary albumin excretion and microalbuminuria.

## Limitations of the study

Our study had some potential limitations. The cross-sectional design limited causal inferences. The mechanisms that link severity of obesity and increased urinary albumin excretion remained unclear. We used BMI instead of waist-hip ratio to assess abdominal obesity. Therefore, we cannot exclude the possibility of residual confounding and finally we ignored assessing dietary patterns of the individuals might affect relationship between severity of obesity and microalbuminuria.

## Acknowledgments

This manuscript is the presentation of the fellowship thesis and has been supported by Tehran University of Medical Sciences. We thank Tehran University of Medical Sciences for their support.

## Authors’ contribution

FM: principal author, corresponding author, scientific writing, coordinator. MMM: principal co-author, statistical analysis. MRA: second co-author, help in scientific writing. SS; detecting patients, second coordinator.

## Conflicts of interest

The authors declare no conflict of interests.

## Ethical considerations

Ethical issues (including plagiarism, misconduct, data fabrication, falsification, double publication or submission, redundancy) have been completely observed by the authors.

## Funding/Support

This manuscript is the presentation of the nephrology fellowship thesis and has been supported by Tehran University of Medical Sciences that provided funding for this research (grant no. PB374).
